# SIK2 attenuates proliferation and survival of breast cancer cells with simultaneous perturbation of MAPK and PI3K/Akt pathways

**DOI:** 10.18632/oncotarget.25082

**Published:** 2018-04-24

**Authors:** Neslihan Zohrap, Özge Saatci, Burcak Ozes, Ipek Coban, Hasan Murat Atay, Esra Battaloglu, Özgür Şahin, Kuyas Bugra

**Affiliations:** ^1^ Department of Molecular Biology and Genetics, Bogazici University, Istanbul, Turkey; ^2^ Department of Molecular Biology and Genetics, Bilkent University, Ankara, Turkey; ^3^ Department of Pathology, Istanbul Florence-Nightingale Hospital, Istanbul, Turkey; ^4^ Department of General Surgery, Gayrettepe Florence-Nightingale Hospital, Istanbul, Turkey; ^5^ Life Sciences Center, Bogazici University, Istanbul, Turkey

**Keywords:** SIK2, tumor suppressor, breast cancer, Ras/ERK and PI3K/Akt signaling, EMT

## Abstract

Salt Inducible Kinase2 (SIK2) has been shown to contribute to tumorigenesis in multiple tumor types in a dichotomous manner. However, little is known about its contribution to breast malignancies. Here, we report SIK2 as a potential tumor suppressor in breast cancer whose expression was reduced in tumor tissues and breast cancer cell lines compared to normal counterparts. *In vitro* loss- and gain-of-function experiments combined with xenograft studies demonstrated that SIK2-mediated attenuation of proliferation and survival of breast cancer cells with parallel inhibition of both Ras/Erk and PI3K/Akt pathways. Our findings elucidated that SIK2 has also an inhibitory role in migration/invasion ability of breast cancer cells through regulation of epithelial mesenchymal transition. Immunostaining of patient tumors revealed that SIK2 protein level is frequently downregulated in invasive mammary carcinomas and negatively correlated with the mitotic activity of the cells in triple negative breast cancers and hormone positive tumors. Strikingly, patient survival analysis indicated that higher levels of SIK2 are significantly associated with better survival, especially in basal breast cancer cases. Overall, our findings suggest SIK2 as a potential tumor suppressor in the control of breast tumorigenesis, at least in part, *via* inhibiting PI3K/Akt and Ras/ERK signaling cascades simultaneously and a novel prognostic marker, especially in basal subtypes of breast cancer.

## INTRODUCTION

Breast cancer is the second leading cause of cancer related deaths among women worldwide. It is a complex disease with inter-/ intra-tumor molecular heterogeneity which affects disease progression and treatment responses [1–2]. Breast cancer classification based on molecular profiling approaches have contributed to a better understanding of breast cancer biology and led to five major subtypes: luminal A/B, basal-like, HER2(+), normal breast-like [3–4]. This allowed for the development of targeted therapies for some specific subtypes, e.g. tamoxifen for luminal-A tumors and trastuzumab for HER2+ subtype. However, acquired drug resistance, metastasis and relapse risk remain major challenges [5, 6]. Therefore, finding novel targets and biomarkers is needed for better management of the disease, especially for basal breast cancers for which no efficient therapy options available.

Signaling pathways activated *via* RTKs control essential cellular events such as proliferation, survival, migration and differentiation [7]. Gain of function mutations in a number of RTKs and their ligands as well as the hyper-activation of major downstream signaling cascades Ras/ERK and PI3K/Akt play critical roles in various cancers including breast malignancies [8]. While hyper-activation of ERK1/2 inducing MAPK cascade has been reported in nearly 50% of breast tumors, aberrant activity of PI3K/Akt signaling is observed in 70% of all breast adenocarcinomas [9, 10]. Accumulating evidence also points to genetic and epigenetic aberrations in the negative regulators of these pathways [11, 12]. Loss of function alterations have been shown for negative modulators of MAPK/ERK pathway such as DUSP4, NF1, SPRY and Sef [13–16], while reduced expression of PTEN tumor-suppressor as essential regulator of PI3K/AKT signaling has been documented in 33% of breast tumors [17]. Down-regulation of TSC1/TSC2 tumor suppressors which control PI3K/Akt signaling is closely associated with the development of metastatic breast cancers [18]. Furthermore, the loss of specific miRNAs targeting the transducers of these pathways is also linked to poor prognosis with enhanced metastatic risk and drug resistance [19–20]. In this study, we introduce SIK2 as a novel potential tumor suppressor in breast cancer progression mediating its effects, in part, *via* simultaneous inhibition of Ras/ERK and PI3K/Akt signaling pathways.

SIK2, Ser/Thr kinase, has been implicated in diverse biological processes such as regulation of insulin signaling [21, 22], gluconeogenesis [23], melanogenesis [24], neuronal survival [25], control of tissue size [26], and autophagy [27]. Some reports suggest an oncogenic role of SIK2 in different tumor types. The first report demonstrating SIK2 in tumorigenesis was in the context of ovarian cancer, where it was suggested to promote G2/M transition and centrosome separation [28]. In prostate cancer SIK2 was shown to inhibit apoptotic cell death through the regulation of CREB1-dependent gene regulation [29]. In the same line, SIK2 was associated with the survival of glioma cells through suppression of S6K [30]. Silencing of USP1 was shown to target SIK2 to inhibit colony formation capacity and invasiveness of osteosarcoma cells by stimulating apoptosis [31]. Liu group indicated that overexpression of miR-203 sensitized Taxol resistant colorectal cancer cells *via* targeting SIK2 [32]. In another study, increased autophagic flux into TNBC cells was achieved by blocking of SIK2 [33]. Miranda and colleagues demonstrated that SIK2 contributes to ovarian tumor metastasis by localizing to the adipocyte rich environment to support survival and metabolic requirements of the cells [34]. In contrast to its tumor promoting roles, SIK2 was also suggested to suppress metastasis and contributed to patient survival in renal and liver cancers [35]. Interestingly, in a cohort of breast cancer patient, deletion of genomic region 11q23, which includes the SIK2 gene, was reported [36]. Taken together, these studies point towards a possible dichotomous role of SIK2 in cancer progression and metastasis.

Here, we report the reduced levels of SIK2 expression in breast cancer tissues and in breast cancer cell lines suggesting a tumor suppressor role for SIK2 in breast cancer. *In vitro* loss-of-function and gain-of-function experiments combined with xenograft studies demonstrate that SIK2 attenuates proliferation and survival responses of breast cancer cells with concomitant inhibition of Ras/Erk and PI3K/Akt pathways. Furthermore, SIK2 functions as an inhibitor of migration and invasion of breast cancer cells through regulation of EMT. Analysis of patient specimen and clinical data showed that SIK2 expression is downregulated in breast tumors and associated with patients’ survival, especially for basal subtype, suggesting its prognostic potential.

## RESULTS

### SIK2 expression is downregulated in breast cancer tissues and cell lines

**Figure 1:**
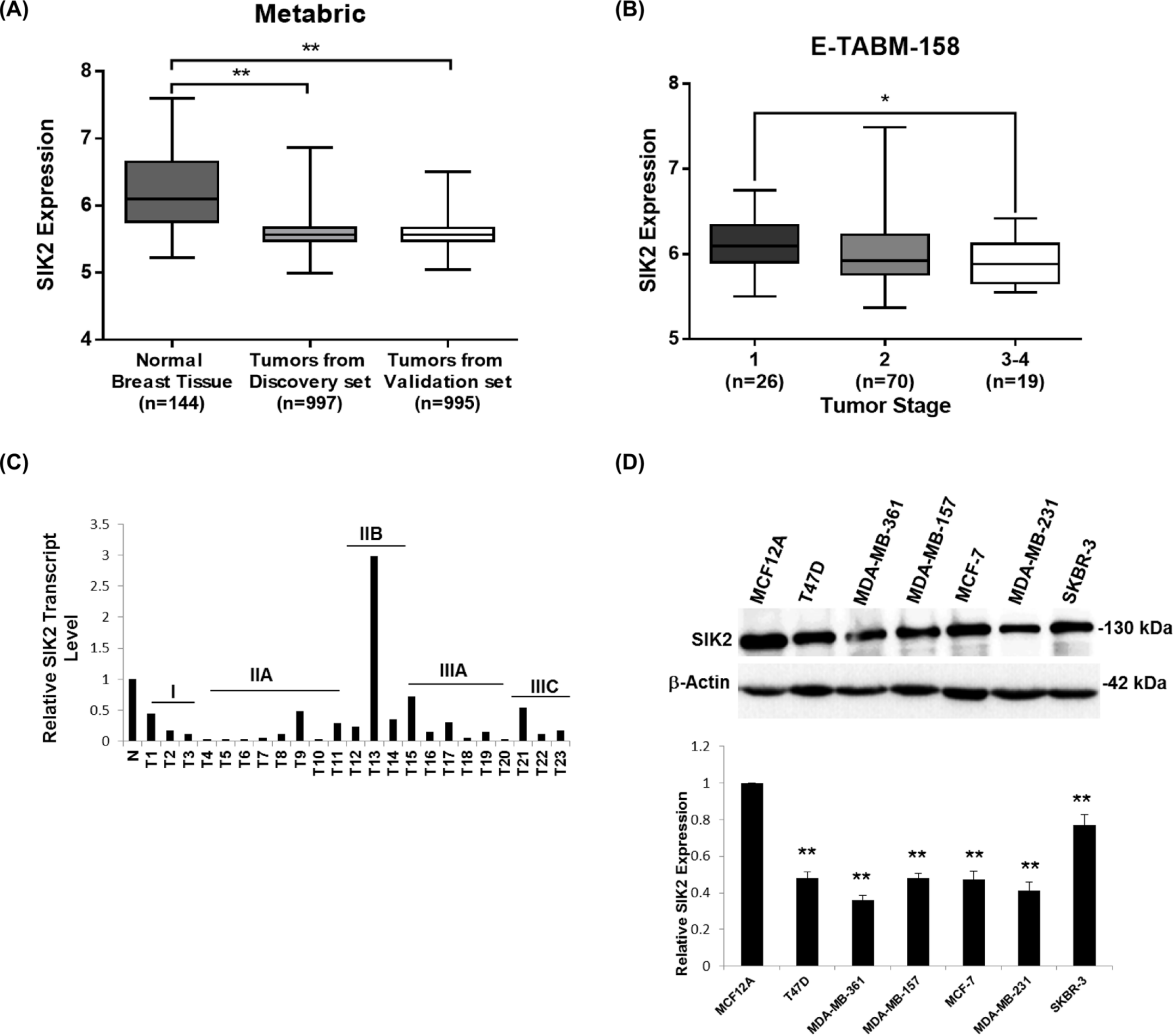
SIK2 expression is downregulated in breast cancer tissues and cell lines SIK2 expression levels were analyzed **(A)** in breast tumors as compared to normal tissues using discovery and validation sets from the METABRIC data and **(B)** in late and early stage breast tumors using E-TABM-158 RNA sequencing dataset. **(C)** q-RT-PCR was performed using a commercial breast cancer cDNA array. SIK2 levels were normalized to that of GAPDH in the same sample. N represents the mean value of SIK2 transcript levels from two independent normal breast tissue samples; T1-T23 correspond to the individual primary tumors of grades I to IIIC. **(D)** Western blot analysis of lysates from indicated breast cancer cell lines was performed. Bar graphs represent mean value of five independent biological samples after normalization of SIK2 band intensities to that of β-actin. Statistical significance was assessed by unpaired, two-tailed student t-test. (^**^P<0.001, and ^*^P<0.05).

To establish a potential link between SIK2 expression and breast malignancies, we first investigated SIK2 expression profiles of breast cancer tissues in comparison to normal mammary gland using publicly available RNA sequencing and microarray profiling datasets. The METABRIC (EGAS00000000083) dataset [37] which comprises clinic-pathology data from discovery (n=997) and validation sets of primary breast tumors (n=995), revealed a significantly lower SIK2 expression in breast cancer tissues than in normal samples (n=144) (Figure 1A). Similarly, 9 out of 13 independent datasets retrieved from ONCOMINE database (www.oncomine.org) [38] showed statistically significant downregulation of SIK2 level in breast tumor samples as compared to normal tissues (Supplementary Table 1A). Interestingly, query into GSE53752 microarray dataset [39] indicated significantly lower SIK2 expression in TNBC patients with IDC (n=51) (Supplementary Figure 1). E-TABM RNA sequencing dataset [40] showed a statistically significant reduction of SIK2 expression in advanced stage of breast tumors (n=19, stage 3-4) as compared to early tumor stages (n=26; stage 1; Figure 1B). Consistent with the expression data, frequent loss of SIK2 copy number was seen in breast tumor samples of the ‘TCGA Breast 2’ dataset, which comprises 759 tumor samples and 813 normal tissues (www.oncomine.org) (Supplementary Table 1B). We then analyzed SIK2 transcript levels directly by q-RTPCR using a cDNA array which included 23 tumor samples ranging from stage I to IIIC and 2 normal breast samples for the comparison. With the exception of a single stage IIB sample, all other samples showed a reduced SIK2 transcript level that varied between 20-80% of SIK2 expression in normal tissues (Figure 1C).

Next, we asked if SIK2 may also be downregulated in breast cancer cell lines when compared to normal cell line. In line with our expectation, T47D, MDA-MB-361, MDA-MB-157, MCF-7, MDA-MB-231, and SKBR-3 cell lines showed reduced protein expression in comparison to basal MCF12A cells derived from normal breast epithelium (Figure 1D). Thus, our analysis supports the notion that SIK2 expression is frequently downregulated in primary breast cancer samples and breast cancer derived cell lines.

### SIK2 inhibits proliferation and survival of breast cells with parallel downregulation of MAPK and PI3K/Akt signaling pathways

**Figure 2:**
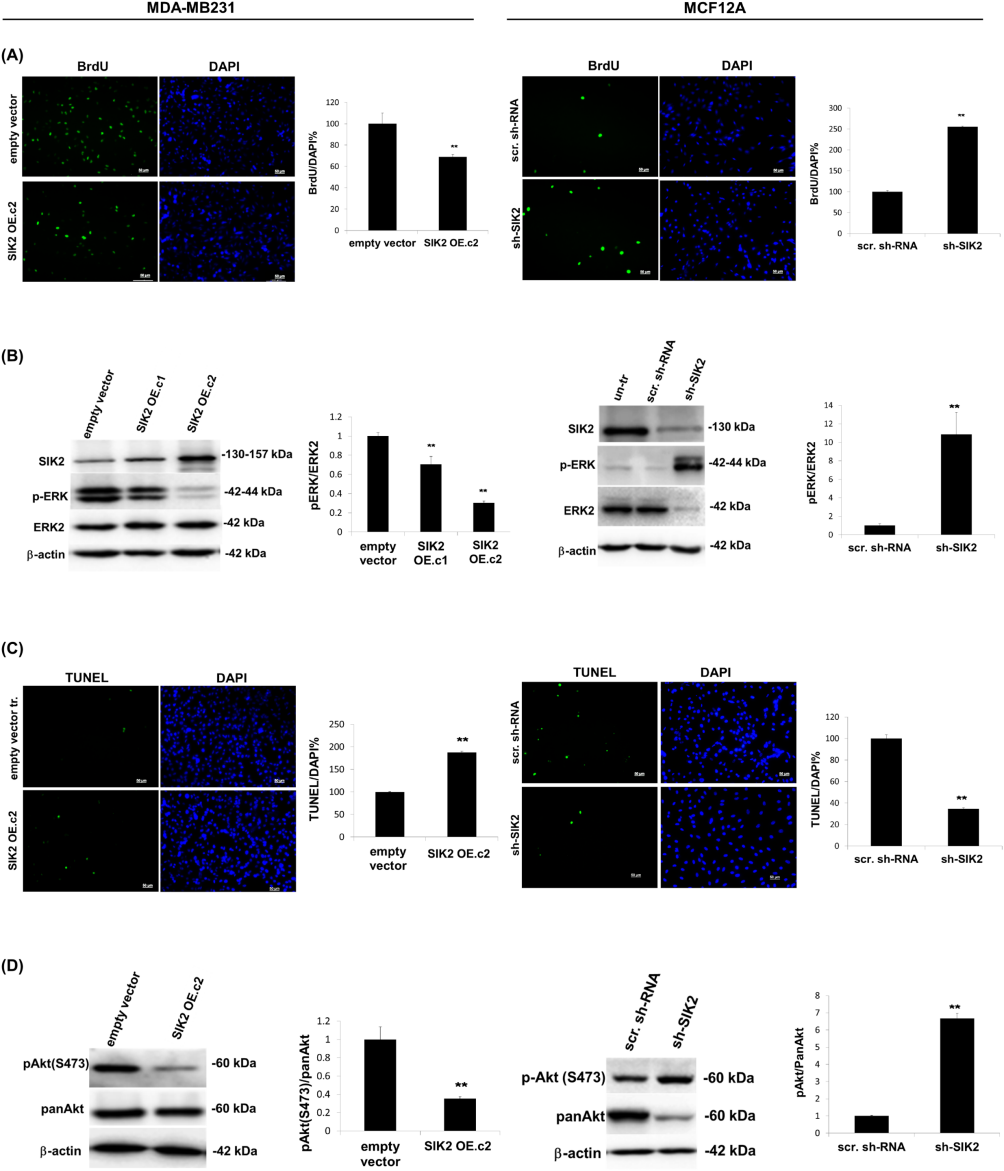
SIK2 inhibits proliferation and survival of breast cells with parallel downregulation of MAPK and PI3K/Akt signaling pathways SIK2 expression was modulated in MDA-MB-231 cells by transfection with full-length SIK2, and in MCF12A cells by transduction with lentivirus carrying sh-SIK2. Empty vector transfection or scrambled sh-RNA transduction constituted the controls, respectively. The left panels represent data from MDA-MB-231 and the right panels from MCF12A cell lines. **(A)** Proliferation was assessed by BrdU incorporation assay subsequent to FGF2 (1 ng/ml) treatment of cells, serum starved overnight. **(B)** Changes in active Erk levels in two different clones of SIK2 OE cells; expressing low (SIK2 OE. c1) and high (SIK2 OE. c2) levels of SIK2, compared to empty vector-transfected cells were evaluated by Western blotting. p-ERK band intensities were normalized to that of ERK in the same samples. SIK2 OE. c2 was used for further overexpression studies. **(C)** TUNEL assay was performed on cells that were serum starved overnight. **(D)** Changes in active Akt levels in serum starved, SIK2 OE cells as compared to empty vector-transfected cells by Western blotting. p-Akt band intensities were normalized to that of pan-Akt in the same samples. Bar graphs represent the mean values of at least 3 independent biological samples (± SD). In BrdU and TUNEL assays, cell nuclei were visualized by DAPI staining and in each sample at least 200 cells were counted. ^**^P < 0.001 and the Scale bar=50um.

To investigate whether SIK2 has an effect on proliferation and survival of breast cells, SIK2 expression was stably upregulated in highly tumorigenic MDA-MB-231 cells by transfection with a plasmid encoding the full-length coding sequence. For loss-of-function analysis, the non-transformed MCF12A cell line was used to prepare clones in which SIK2 was stably downregulated using a shRNA. The overexpression studies indicated that, high SIK2 levels (SIK2 OE. c2) led to approximately 30% decline in proliferation of MDA-MB-231 cells compared to the empty vector-transfected controls (Figure 2A, left panel), and lower SIK2 expression (SIK2 OE. c1) resulted in only 12% growth inhibition (Supplementary Figure 2). This dose-dependent decrease in MDA-MB-231 proliferation was also reflected in active ERK levels (Figure 2B, left panel). When SIK2 was overexpressed in SK-BR-3 cells, a malignant with relatively high endogenous SIK2 line compared to MDA-MB-231, neither p-ERK levels, nor cellular growth were attenuated (Supplementary Figure 3). These data suggest that in cell lines with high endogenous SIK2 such as SK-BR-3 cells, a further increase in SIK2 expression is likely to have no effect on cellular growth, there might be other factors contribute to their aggressive behavior. On the other hand, MCF12A cells with downregulated SIK2 levels showed enhanced BrdU incorporation (2.6-fold of scrambled controls) (Figure 2A, right panel) accompanied by a 10-fold increase in p-ERK levels (Figure 2B, right panel). Furthermore, we observed a nearly 2-fold increase in the fraction of apoptotic cells (Figure 2C, left panel) and significantly lower Akt phosphorylation levels (67%) were observed in the MDA-MB-231 cells with elevated SIK2 level as compared to control (Figure 2D, left panel). Complementary to this, depletion of SIK2 in MCF12A cells led to 64% reduction in apoptotic cell population (Figure 2C, right panel) and nearly 7-fold increase in p-Akt levels compared to the controls (Figure 2D, right panel). Overall, our data suggest that SIK2 inhibits proliferation and survival of breast cells possibly by acting as a dual inhibitor of Ras/Erk and PI3K/Akt signaling cascades.

### SIK2 inhibits migration and invasion of breast cells *via* blocking EMT

**Figure 3:**
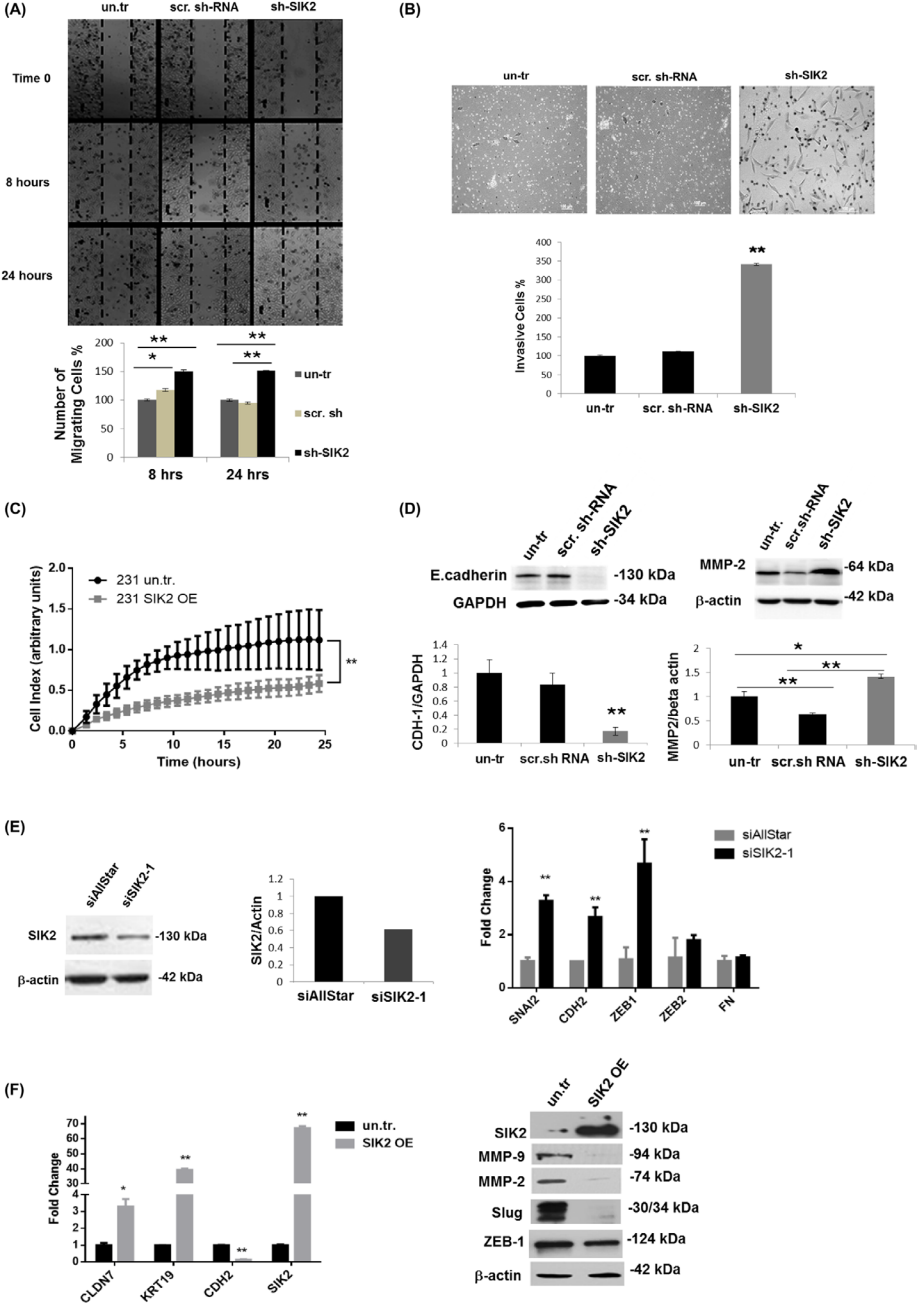
SIK2 inhibits migration and invasion of breast cancer cell lines *via* blocking EMT The effect of SIK2 in the motility and invasiveness behavior of the breast cells was investigated in the cell lines with SIK2 modulation. **(A)** Effect of stable downregulation of SIK2 on the motility of MCF12A cells were evaluated relative to the control cells by wound healing assay. Cells migrated to the scratch area were counted at the indicated time points. **(B)** Potential changes in invasive characteristics of MCF12A cells upon stable SIK2 down-regulation were assessed by matrigel trans-well assay. Filters were collected at 48 hours after the initial seeding; cells were stained with Giemsa and counted. **(C)** Effect of stable upregulation of SIK2 on the migration of MDA-MB-231 cells was analyzed by RTCA analysis in a 24 hours’ time frame. **(D)** Effect of SIK2 downregulation on E-cadherin and MMP2 expression profiles of MCF12A cells was studied by Western blot analysis. **(E)** Efficacy of transient SIK2 silencing by siRNA in MCF12A cells was assessed by Western blotting and its impact on the expression of indicated EMT markers was compared to control cells (siAllStar) by qRT-PCR. **(F)** Effect of SIK2 upregulation on indicated EMT markers in MDA-MB-231 cells was evaluated by qRT-PCR. In Western blotting either anti-β-actin or anti-GAPDH antibody was used for loading control, HPRT and GAPDH were used as the internal controls in qRT-PCR. All experiments were done in triplicate. ^**^P< 0.001, and ^*^P≤ 0.05.

Next, we tested whether SIK2 has an influence on metastatic behavior of breast cells and, therefore, examined cell migration by wound healing and Matrigel trans-well assays using MCF12A cells with stably reduced SIK2 level. The number of cells closing the wounded area increased 38% within 8 hours, and 50% within 24 hours following the initial wound formation as compared to the controls (Figure 3A). In agreement with the increased motility, a significant increase (3.4-fold) in the number of invasive cells was observed with depleted SIK2 levels as compared to scrambled-sh controls (Figure 3B). Reciprocally, Real Time Cell Analyzer (RTCA) experiment revealed that enhanced SIK2 expression led to a significant reduction in motility of MDA-MB-231 tumorigenic cells in comparison to empty vector-transfected controls (Figure 3C). This approach also demonstrated that the migration capacity of MDA-MB-231 cells is decreasing with enhanced SIK2 levels in a dose-dependent manner (Supplementary Figure 4A).

The above findings prompted us to investigate possible changes in the expression of EMT markers in the same cells. In line with our hypothesis, we observed a strong downregulation of epithelial marker E-Cadherin, while expression of the mesenchymal marker MMP-2 was strongly induced upon SIK2 knockdown (Figure 3D). This observation is further supported by increased expression of additional mesenchymal markers SNAI2, CDH2 and ZEB1 in MCF12A cells upon transient knockdown of SIK2 (Figure 3E). In contrast, stable overexpression of SIK2 in MDA-MB-231 cells led to an increase in the expression of epithelial markers Claudin-7 and KRT-19 while the expression of mesenchymal markers CDH2, MMP-2, MMP-9, Slug and ZEB1 was strongly downregulated (Figure 3F). Furthermore, we observed a dose-dependent decrease in the expression of mesenchymal markers ZEB1 and MMP-9 upon increased expression of SIK2 (Supplementary Figure 4B). Altogether, these findings suggest that SIK2 inhibits migration and invasion in breast cancer cell lines *via* blocking EMT.

### Attenuation of tumor growth by SIK2 is accompanied with simultaneous inhibition of MAPK and PI3K/Akt signaling pathways

**Figure 4:**
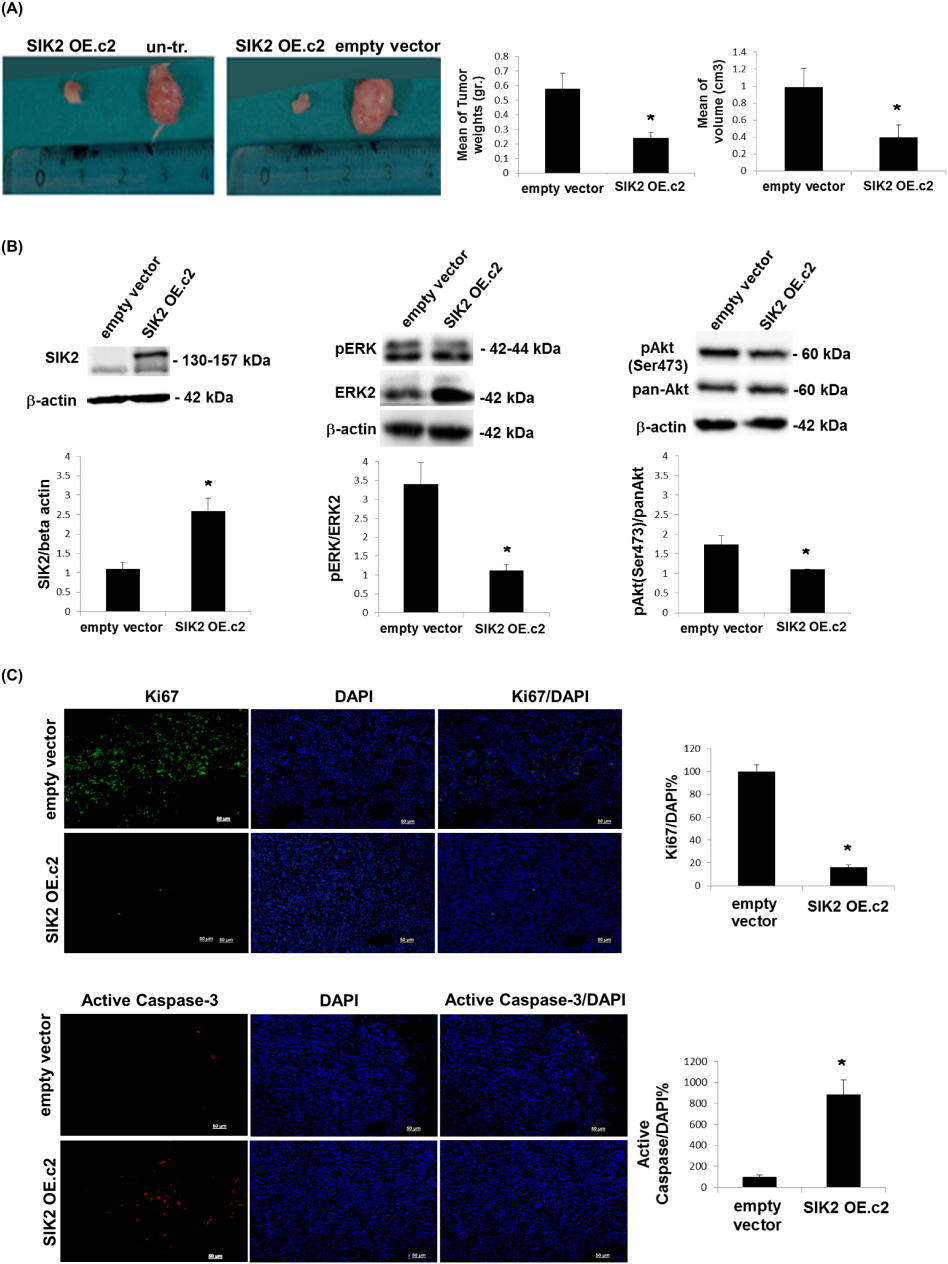
Attenuation of tumor growth by SIK2 is accompanied with simultaneous inhibition of MAPK and PI3K/Akt signaling pathways MDA-MB-231 cells overexpressing SIK2 were injected subcutaneously to left flanks of SCID mice while the right flanks received empty vector transfected or parental controls (n=6 mice per group). **(A)** Representative images of tumors excised 5 weeks post injections were shown. Bar graphs represent mean values of the weights and volumes of tumors (± SD). **(B)** Lysates from excised tumors were subjected to Western blotting. Blots were probed with the indicated antibodies. Band intensities of SIK2 were normalized to that of β-actin, p-ERK to that of ERK and p-Akt to that pan-Akt; mean values of three independent experiments are presented as bar graphs (± SD). **(C)** Tumor sections (6 sections / tumor) were stained with anti-Ki67 or anti-active caspase-3, and nuclei were visualized by DAPI. The fractions of Ki67 or active caspase-3 positive cells were graphically represented. ^*^P≤0.05 and the Scale bar: 50 um.

To test the tumor suppressor potential of SIK2 *in vivo*, MDA-MB-231 cells with enhanced SIK2 levels and empty vector transfected or un-transfected control lines were injected subcutaneously to either flank of SCID mice. When tumor growth was assessed 5 weeks post injection, we observed that MDA-MB-231 cells with elevated SIK2 levels formed significantly smaller tumors in weight and volume as compared to control injections (Figure 4A). An analysis of the excised tumors of the xenografts by immunoblotting indicated that high SIK2 levels were maintained in the cells overexpressing SIK2 (Figure 4B, left panel). Consistent with our *in vitro* findings, a significant decrease in p-ERK levels (Figure 4B, middle panel) and the number of dividing cells, as shown by the mitotic marker Ki67(Figure 4C, top panel) was observed in the tumors that originated from MDA-MB-231 cells with upregulated SIK2 levels. The results confirm our *in vitro* findings (Figure 2A) with respect to the inverse correlation between SIK2 levels and mitotic ability of cells *in vivo* and support our hypothesis that SIK2 activity contributes to the inhibition of breast cancer cell proliferation. Similarly, significantly reduced levels of p-Akt (Figure 4B, right panel) and increased active caspase-3 signal (Figure 4C, bottom panel) were detected in the tumors with SIK2 overexpression. These findings further support the inhibitory role of SIK2 for the survival capacity of breast cancer cells. Therefore, we conclude that SIK2 suppresses tumor progression, at least in part, through the inhibition of proliferation as well as survival capacities of the breast tumor cells, which can conceivably be mediated *via* simultaneous inhibition of Ras/ERK and PI3K/Akt signaling pathways.

### SIK2 protein expression is frequently downregulated in breast tumors and negatively correlated with the mitotic activity of the breast cells

**Figure 5:**
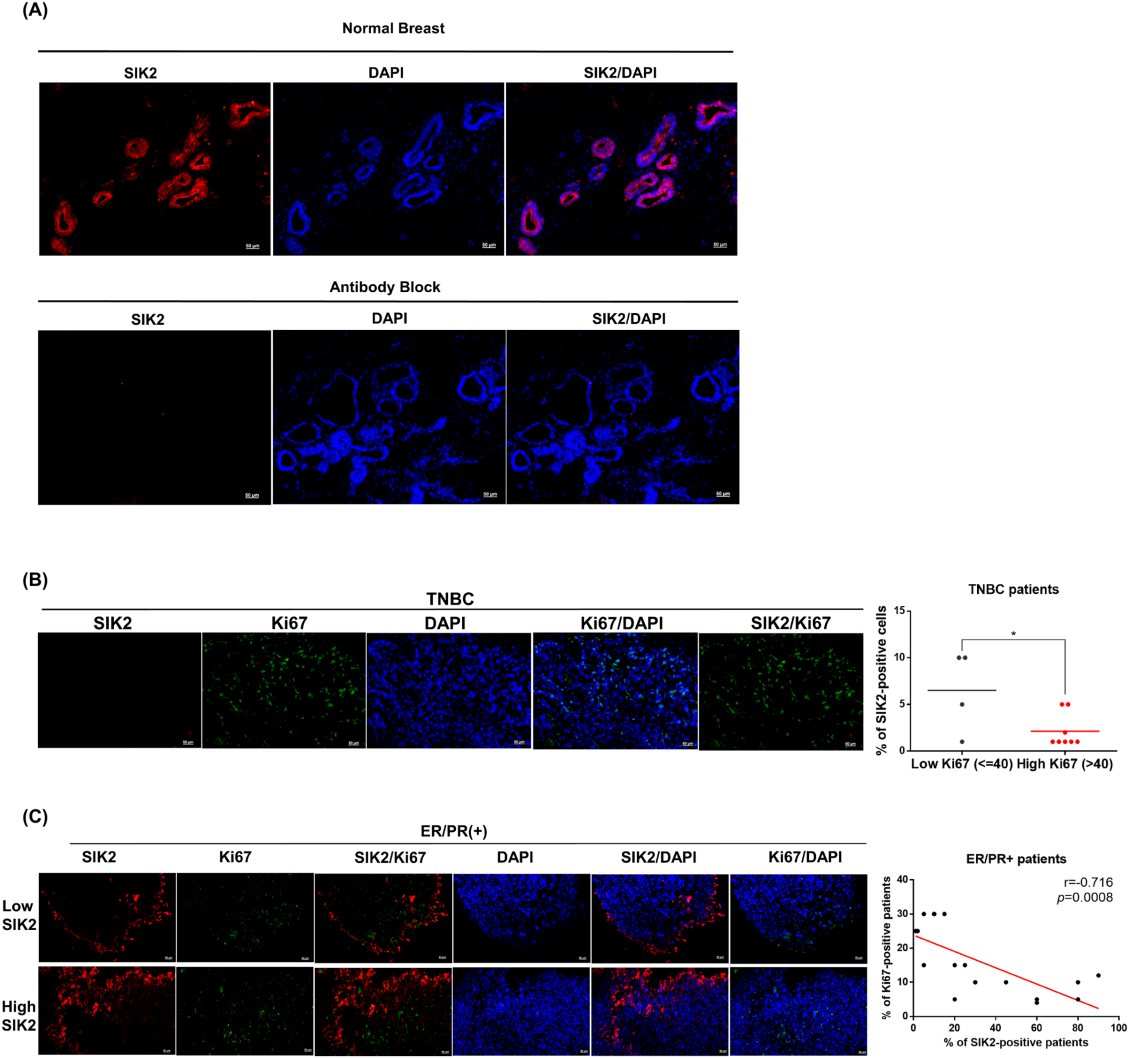
SIK2 protein expression is frequently downregulated in breast tumors and negatively correlated with mitotic activity of the breast cells Tissue sections obtained from breast cancer patients were stained with anti-SIK2 (red), and/or anti-Ki67 (green) antibodies, the nuclei were visualized with DAPI. **(A)** Top panel is a representative image of normal human breast tissue stained with SIK2 antibody. In the bottom panel, the primary antibody was pre-incubated with affinity purified recombinant SIK2 protein. **(B)** Representative image of TNBC tumors co-immunostained with SIK2 and Ki67. SIK2 expression from low and high Ki67 positive samples was compared. **(C)** Co-immunostaining of ER/PR positive tumors with SIK2 and Ki67 antibodies. The scatter plot shows the negative correlation between Ki67 and SIK2 expression. ^*^, *P*<0.05 and scale bar=50um.

Next, we investigated SIK2 protein expression in normal mammary gland and primary tumor samples collected from patients. The clinic-pathological features of all patient samples are summarized in Supplementary Table 2. IHC staining revealed that SIK2 is diffusely (score: +4, >75%) expressed in the ductal epithelial cells of healthy breast tissue (Figure 5A, top panel). No signal was detected when SIK2 antibody was pre-incubated with SIK2 blocking peptide, indicating the specificity of the staining (Figure 5A, bottom panel). We found that no SIK2 signal was detectable in all tumor samples that are classified as TNBC (Figure 5B). Diffuse (score: +4, >75%) SIK2 signal persisted in 8/18 cases of ER/PR (+) tumor samples (Figure 5C, top panel), while the remaining ER/PR (+) tumors showed notably reduced SIK2 expression (score 0: 1 case, score +1: 8 cases, score +2: 1 case) compared to the neighboring non-transformed tumor areas as in the TNBCs (Figure 5C, bottom panel). Strikingly, double-label immunohistochemistry against SIK2 and Ki67 revealed a significant loss of mitotic cells in the regions where persistent SIK2 expression could be observed (Figure 5C, top panel). Conversely, signal for the proliferation marker Ki67 was strongly detectable in ER/PR (+) tumor areas where no SIK2 expression is detectable (Figure 5C, bottom panel). These data confirm that SIK2 protein expression (similar to its RNA levels as show in Figure 1A-1C) is frequently downregulated in different breast tumor subtypes, but its expression is completely lost in TNBCs. Furthermore, inverse correlation between SIK2 and the mitotic marker expression strongly supports our *in vitro* ad *in vivo* findings and suggests an inhibitory role of SIK2 in the breast tumorigenesis.

### High SIK2 expression is associated with better survival in breast cancer patients

**Figure 6:**
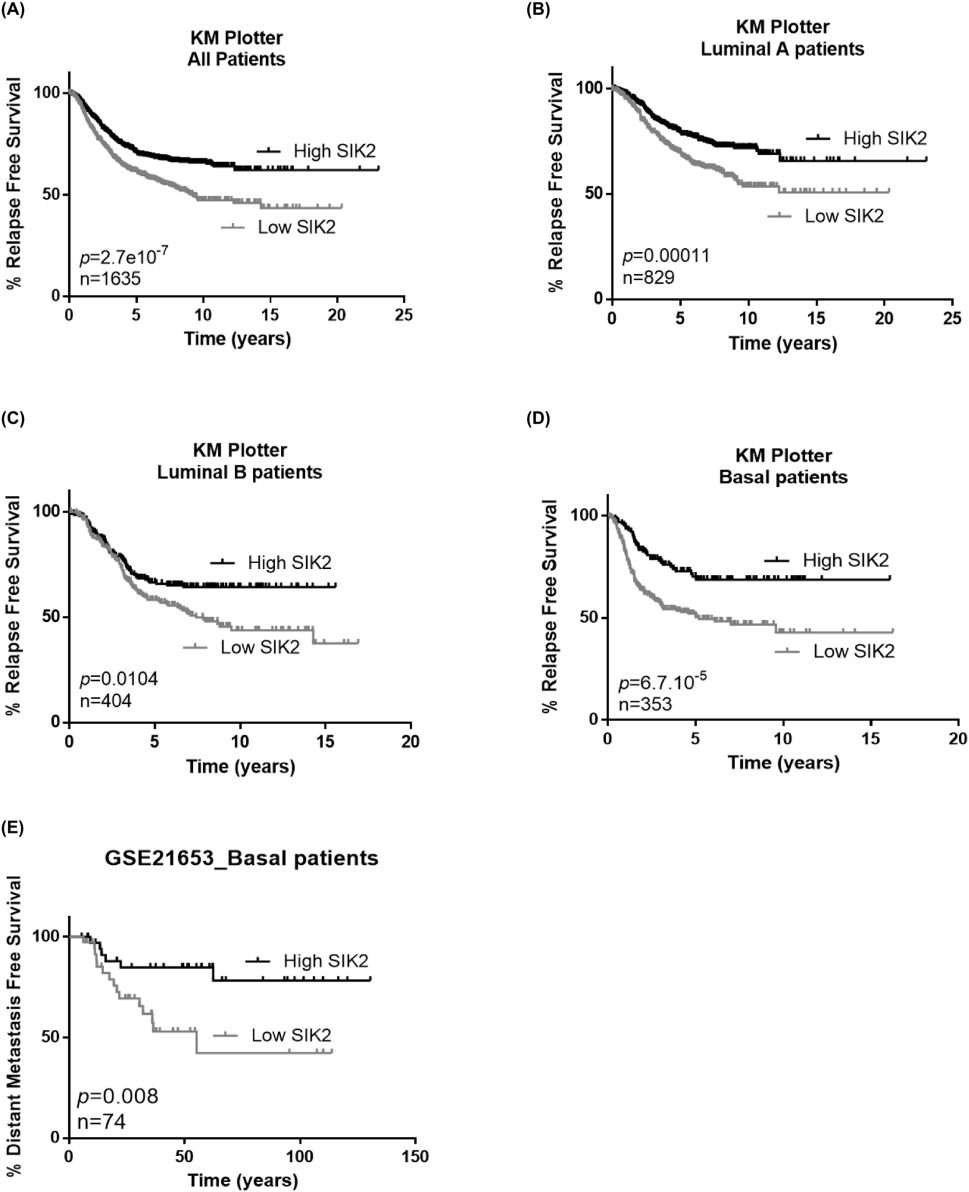
High SIK2 expression is associated with better survival in breast cancer patients To reveal the association between SIK2 expression levels and survival of breast cancer patients Kaplan Meier (KM) plotter analysis was performed in **(A)** all subtypes, **(B)** Luminal A, **(C)** Luminal B **(D)** basal subtype. **(E)** Prediction of disease free survival of basal subtype patients on the basis of median separation of SIK2 expression in GSE21653 dataset. KM plots were generated in GraphPad software. Statistical significance of the separation between the two curves was assesed by Log-rank test.

Since our *in vitro* and *in vivo* data indicated a negative correlation between SIK2 levels and breast cell proliferation and survival, we next asked if SIK2 levels are associated with survival of breast cancer patients. Kaplan-Meier analysis showed that higher SIK2 levels are significantly associated with better RFS in all patients including different subtypes of breast cancer (n=1635) (Figure 6A). When this analysis was carried out specifically for the Luminal A and B subtypes, again we observed that higher SIK2 level revealed better RFS (Figure 6B and 6C, respectively). The association of high SIK2 levels with RFS was more significant for basal cancer patients (n=353; p=6.7×10^-5^) compared to the Luminal A and Luminal B subtypes (Figure 6D), [41]. Analysis of an independent dataset, GSE21653, showed that high levels of SIK2 is significantly associated with DFS in basal patients (n=74; p=0.008) (Figure 6E) [42]. Overall, these data suggest SIK2 expression as a prognostic marker in breast cancer, especially for basal subtype.

## DISCUSSION

Ras/ERK and PI3K/Akt pathways are triggered by diverse ligands and frequently hyper-activated in human breast cancers. Loss-of-function changes in negative regulators of these cascades contribute to disease progression, which is closely associated with poor prognosis and resistance to targeted therapies [43]. In this study, we report that SIK2 is implicated in simultaneous inhibition of Ras/ERK and PI3K/Akt signaling pathways to attenuate proliferation, survival and motility of the breast cells. Based on our *in vitro* and *in vivo* functional analyses combined with patients’ tumor analyses, we propose SIK2 as a novel tumor suppressor and a potential biomarker for breast cancer.

The first line of evidence suggesting a possible tumor suppressor function of SIK2 in breast cancer came from queries into RNA sequencing and microarray profiling data which demonstrated a frequent down regulation of SIK2 expression in primary breast tumors of various subtypes as compared to normal mammary gland. In agreement with these results, a significant decline in SIK2 levels was observed in a set of breast cancer lines compared to non-transformed MCF12A cells. The enhanced SIK2 expression led to decreased proliferation and survival of highly tumorigenic MDA-MB-231 cells accompanied with reduced activation in Erk and Akt effector kinases. Reciprocally, knockdown of SIK2 in basal cell line MCF12A resulted in enhanced proliferation and survival accompanied by upregulation of p-ERK and p-Akt levels. Moreover, MDA-MB-231 cells with elevated SIK2 expression generated significantly smaller tumors in xenografts compared to control injections. Thus, we conclude that aberrant loss of SIK2 may contribute to hyper-activation of Ras/ERK and PI3K/Akt signaling pathways, thereby, takes part in tumor development *via* perturbing the delicate balance between proliferation and survival of breast cells. Although we have shown that inhibitory effects of SIK2 on these tumor-related processes e.g., proliferation and invasion, may involve ERK and Akt pathways as potential downstream mediators, further rescue experiments involving modulation of SIK2 and perturbation of these pathways are required to validate SIK2 as a key regulator of these pathways contributing to the observed phenotypic effects. In this study, we did not address the mechanisms by which SIK2 downregulates these cascades. Our earlier work suggested that SIK2 negatively modulates insulin dependent survival of retinal Müller cells through downregulation of IRS-1 [22]. We also have evidence that SIK2 is involved in the negative regulation of FGF2-dependent ERK activation mediated via Gab1 targeting (*Kuser-Abali et al., unpublished data*). Both of these docking proteins are critical effectors of various RTK pathways such as EGFR and IR, which are also implicated in the development of breast malignancies [44–45]. Therefore; one can speculate that deregulation of SIK2 may contribute to tumorigenesis associated with an array of signaling networks.

PI3K/Akt and Ras/ERK pathways are known to contribute to EMT and invasive phenotype of breast cells. For example, enhanced activity of PI3K/Akt pathway was shown to mediate migration of cells through activation of MMP cascade [46]. Sustained activation of PI3K/Akt and MAPK/ERK pathways together increase the expression of EMT regulators Slug and ZEB1 leading to downregulation of E-cadherin and modulating epithelial cell plasticity to support metastasis [47]. In our study, silencing of SIK2 induced motility and invasiveness of MCF12A cells, and upregulation prevented the migration of MDA-MB-231 mesenchymal cells. Further analysis of tumorigenic cells with elevated SIK2 level showed that increased expression of epithelial markers Claudin-7 and KRT-19 is correlated with downregulation of Slug as their common upstream effector protein [48, 49]. In line with this, silencing of SIK2 in MCF12A cells resulted in significant upregulation of SNAI2, CDH2 and ZEB-1 leading to suppression of E-cadherin to promote EMT. Furthermore, we also observed that expression of matrix metalloproteinases MMP-2 and MMP-9 are modulated by SIK2 level. Therefore, in breast tumors, we suggest that SIK2 may function as a metastasis inhibitor *via* simultaneous blockage of Ras/ERK and PI3K/Akt pathways.

In this report, for the first time, we showed that SIK2 is expressed in the ductal epithelial cells of mammary gland and its level was reduced in highly tumorigenic and metastatic TNBC tumors. While in some ER/PR (+) tumors, there was diffuse SIK2 expression similar to their non-tumorigenic tissue counterparts; in others SIK2 expression was limited. Strikingly, mitotic activity was high in tumor regions in which SIK2 is lost. This inverse correlation is in agreement with our *in vitro* findings suggesting SIK2 is a negative regulator of proliferation in the context of breast cells and it acts as a potential tumor suppressor in breast cancer progression. Consistent with this notion, our bioinformatics analysis revealed a close association between higher SIK2 level and better patient survival in luminal A and B subtypes and particularly in basal tumors. Therefore; high SIK2 could be a marker for better prognosis in these subtypes. On the other hand, we did not observe any significant survival difference between HER2+ patients with low or high SIK2 expression. Recently, SIK2 was associated with better survival in renal and liver cancer patients [35]. In contrast, SIK2 was proposed to support survival of TNBC cells with claudin-low subtype via limiting autophagic influx [33]. Since SIK2 was previously suggested as an important modulator of autophagosome maturation [27], its role in cancer cell survival through regulating autophagy requires further investigations.

Although our study suggests SIK2 as a potential novel biomarker in breast tumorigenesis, the mechanisms underlying the reduced SIK2 level remains to be elucidated. Reports of a deletion in 11q23, where SIK2 gene is localized, in a breast cancer patient cohort [36] and significant loss of SIK2 copy numbers in TCGA (2) dataset query [38] are in line with reduced SIK2 expression in breast tumors.

It is important to note that there are also reports that suggest SIK2 as a potential oncogenic marker in ovarian [27, 34], prostate [29], osteosarcoma [31] and colorectal [32] cases via regulating different cellular mechanisms such as CREB-dependent regulation of gene expression, miRNA regulation and cell cycle progression. It is conceivable that aberrations in SIK2 expression/function may result in divergent phenotypes due to the differences in pathways involved, variations in repertoire of interacting partners or existing mutational signatures in specific cellular contexts. Taken together, these findings point to the dichotomous role of SIK2 in tumorigenicity; requiring more detailed investigations. Here, we provide, to the best of our knowledge, the first evidence of SIK2 downregulation, its contribution to breast cancer patient survival, and its potential tumor suppressor role in breast cancer cells, which can potentially be mediated by simultaneous blockage of Ras/ERK and PI3K/Akt pathways. Further studies into the mechanisms of SIK2 loss will contribute to better understanding of neoplastic transformation in breast tissue and designing of new therapeutic strategies.

## MATERIALS AND METHODS

### Human breast tissues

The study was approved independently by the Ethics Committees of Bogazici University and the Florence Nightingale Hospital (2013); all participants provided written informed consent. A total of 30 female patients underwent curative surgical treatment at the Florence Nightingale Hospital (Istanbul, Turkey) from September, 2013 to December, 2014 were included in this study. Tumor and normal tissue areas were randomly selected. Healthy mammary tissue samples were obtained from 3 female subjects undergoing plastic surgery with no history of malignancy. Pathology reports of these patients revealed that 18 of the cases were ER/PR (+) and 12 as TNBC. After excision specimens were fixed in 10% formalin and embedded in paraffin, remaining tissue samples were fresh frozen and stored at -80°C until processed for immunofluorescence staining.

### Cell culture

The cell lines included in this study were kindly provided by Mehmet Ozturk (Izmir Biomedicine and Genome Center) and Isik Yulug (Bilkent University, Department of Molecular Biology and Genetics). All cell culture media and chemicals were purchased from Sigma-Aldrich, USA unless stated otherwise. Non-transformed basal cell line MCF12A was maintained in a 1:1 mixture of DMEM/F12 medium supplemented with 20 ng/ml EGF, 1% NEAA (Biochrom, Berlin, Germany), 0.01 mg/ml human insulin, 500 ng/ml hydrocortisone, 1% Pen/Str and 10% FBS (Thermo Fisher Scientific, USA); MCF-7, MDA-MB-231, MDA-MB-453, MDA-MB-157, MDA-MB-361 cells were cultured in Dulbecco’s modified Eagle’s medium (DMEM) supplemented with 10% FBS, 1% NEAA and 1% penicillin/streptomycin. T47D cells were cultured in RPMI-1640 and SKBR-3 cells were maintained in Mc’Coys medium supplemented with %10 FBS, 1% NEAA and 1% Pen/Str.

### Transfections and lentiviral transduction

For stable silencing of SIK2, MCF12A cells were subjected to lentiviral infection using a pool of lentiviral particles carrying three target specific shRNA (Santa Cruz Biotechnology, USA) as instructed by the manufacturer. The control cells received scrambled sh-RNA (Santa Cruz Biotechnology, USA). The cells were propagated in the presence of 10 μg/ml puromycin (1 mg/ml; Sigma-Aldrich, USA) for 3 weeks. Mixed colonies were collected from at least 4 independent 6-well plates, downregulation was verified by immunoblotting. For transient downregulation, two different siRNAs (Dharmacon, Lafayette, CO, USA) were used with 20 nM final concentration. The siRNA transfections were done using Lipofectamine 2000 (Invitrogen, USA) and Opti-MEM medium (Thermo Fisher, USA). After 48 hours, cell lysates were collected for immunoblotting, RNA isolation and qRT-PCR analysis. For overexpression purposes, a p-EGFP plasmid construct containing full coding sequence of SIK2 was used [23]. MDA-MB-231 cells were transfected using X-treme gene transfection reagent (Roche, Basel, Switzerland). Empty vector transfections were used as control. To select the stable clones, transfected cells were incubated in a medium containing 500 mg/ml G418 (Sigma-Aldrich, USA) for 3 weeks; the media was refreshed in every second days. Each of the two clones, SIK2 OE. c1 and SIK2 OE. c2, with enhanced SIK2 levels were derived from independent single isolated cells. The clone with higher SIK2 expression (SIK2 OE. c2) was used throughout the study unless otherwise stated in the text.

### Quantitative RT-PCR (qRT-PCR)

For analysis of cell lines with transient transfection, total RNA was isolated using TRISure (Bioline, Luckenwalde, Germany) according to the manufacturer’s protocol. For cDNA synthesis, total RNA was reverse-transcribed using Revert-Aid RT Reverse Transcription kit (Life Technologies, USA). Real-time PCR assay was performed using Light Cycler® 480 SYBR Green I Master kit (Roche, Basel, Switzerland); ACTB and HPRT were used as housekeeping genes. The data was analyzed according by ΔΔ*CT* method [50].

For comparative analysis of SIK2 transcript levels in a commercial cDNA array (OriGene, Rockville, MD, USA) including 23 breast tumor samples and 2 normal mammary tissues, SYBR green based q-RTPCR analysis was carried out. GAPDH was used as an internal control in each reaction. Following an initial denaturation step at 95°C for 10 seconds, 40 cycles of amplification was done where each cycle included a denaturation step at 95°C for 10 seconds, an annealing step at 59.5°C for 10 seconds, an extension step at 72°C for 15 seconds. Subsequently, melting curve analysis was carried out and the data was analyzed using BioRAD IQ5 software program. All primer pairs used in these experimental settings were listed in Supplementary Table 3.

### Immunostaining

For routine pathology evaluations 4 μm paraffin sections were stained with hemotoxylin/eosin or processed for IHC staining using an automated stainer (BOND-MAX Automated IHC/ISH Stainer, Germany). For immunofluorescence staining, 8-10 μm cryosections were prepared from primary human and xenograft tumors with their normal tissue counterparts. Briefly, the sections were fixed in 4% PFA and blocked in TBS containing 0.3% Triton™ X-100, 10% normal donkey serum for 2 hours at room temperature. Subsequently, they were incubated with the appropriate primary antibodies overnight at 4°C. Following the extensive washing steps (3 times, 10 minutes each), the appropriate secondary antibodies were applied for 2 hours. Cell nuclei were stained with DAPI. The images were captured using Zeiss Axio-vision Z1 Inverted Fluorescent Microscope (USA) and Leica Confocal Microscope (DM 6000 CS, Germany) equipped with LAF software (LAS AF; Leica, Wetzlar, Germany). Images were optimized for color, brightness, and contrast; double-labelled images overlaid by using Adobe Photoshop 6.0 (Adobe Systems, Inc., New York, NY). All control and test images were digitally enhanced in identical manner. To validate the specificity of SIK2 staining in the tissues, peptide competition assay was carried out. Briefly, SIK2 antibody which was diluted (1/1000) in blocking solution was divided into two tubes; one tube included the blocking peptide at a ratio 5:1 of SIK2 antibody and the second tube had the equivalent volume of PBS instead of blocking peptide. These mixtures were separately incubated overnight at 4°C and staining protocol was applied. All antibodies used in IF staining experiments were listed in Supplementary Table 4. In patient tissue staining, the extent of SIK2 staining was scored as indicated:

0= no signal, 1= less than 10%, 2=10%-50%, 3=50-75% and 4= more than 75% of cells have the signal for SIK2.

### Western blotting

Cell and tissue lysates were prepared in RIPA lysis buffer containing NaCl (1,5M), NP40 (10%), deoxycholicacid (2.5%), 10% SDS, Tris-HCl (0.5 M, pH: 7.4), and protease and phosphatase inhibitors (Roche, Basel, Switzerland). 20 μg of total protein was loaded and separated by sodium dodecyl sulfate-polyacrylamide (SDS-PAGE) gel electrophoresis, transferred to PVDF membrane and probed with primary antibodies overnight at 4°C. After extensive washing steps (3 times, 10 minutes each) with TBST, membranes were incubated with the suitable secondary antibodies. Blots were developed using Amersham Chemiluminescence kit (GE Health Lifesciences, USA). All antibodies used in immunoblotting experiments were listed in Supplementary Table 4.

### Cell proliferation assay

Proliferation was evaluated using *In Situ* Cell Proliferation assay kit (Roche, Basel, Switzerland) per the manufacturer’s instructions. Briefly, cells were seeded on 24-well plates at a density of 100x10^3^cells/mm^2^, and subsequent to serum starvation overnight they were induced with 1 ng/ml FGF2 (R&D Minneapolis, USA) for 24 hour. After extensive washing steps with PBS, the cells were incubated in a medium containing 10 μM BrdU for 5 hours. After removal of BrdU labelling solution, cells were incubated in blocking solution for 1 hour and treated with anti BrdU Fluos antibody. Cell nuclei were stained with DAPI. Samples from three independent experiments were examined using Zeiss Axio Observer Z1 Inverted fluorescent microscope (USA). 200-300 cells were counted for each image taken from at least 6 independent areas per sample.

### TUNEL assay

Cells were seeded on coverslips in a 24-well plate at a density of 100x10^3^cells/mm^2^. After an overnight starvation, cell death was examined by TUNEL assay using the *In Situ* Cell Death kit (Roche, Basel, Switzerland) as per manufacturer’s instructions. To visualize the nuclei, cells were incubated with DAPI for 5 minutes. Samples from three independent experiments were evaluated by Zeiss Axio Observer Z1 Inverted fluorescent microscope (USA). 200-300 cells were counted for each image taken from at least 6 independent areas per sample.

### Cell migration assays

For wound healing assay, confluent cultures were serum starved overnight; a scratch was generated using a 200 μl pipette tip. After washing with PBS, cells were incubated in a medium containing 1% FBS. The photos of the same areas were taken at initial wound formation and following 8 and 24 hours under a microscope (Zeiss Axio Vision Z1 Inverted Microscope, USA). 3 independent experiments were carried out. Cells in the scratch area were counted and mean values of the cell percentages were graphically represented. Real-time migration assay (RTCA) was done using xCELLigence RTCA-DP instrument (ACEA Biosciences Inc., CA, USA) according to manufacturer’s protocol. Briefly, in each well of the bottom chamber of CIM plate, DMEM with 10% FBS was put, while top chambers included DMEM with 1% FBS. 40x10^3^ cells were seeded in the wells of top chamber. Experiment was done in triplicate for each sample. The measurements were taken in every 1 hour; and each sweep point was shown as a dot on the line graph. All graphical representations and statistical analysis was done using GraphPad software (GraphPad software Inc., La Jolla, CA, USA).

### Trans-well invasion assay

Invasion assay was performed using matrigel (Corning, USA) coated trans-well membrane inserts, 8-μm pores with 2x10^4^ pore density (BD Biosciences Durham, USA) according to the manufacturer’s protocol. Briefly, after an overnight starvation, 250x10^3^ cells were layered to the top chamber in a medium devoid of serum. The chambers were set into 24-well plates containing medium supplemented with 10% bovine serum albumin. Cells were allowed to migrate for 48 hours at 37°C. The inserts were fixed in 4% PFA for 10 minutes, washed with PBS and incubated with methanol for 30 minutes. Giemsa staining was carried for 1 hour. After gentle removal of the cells remained in the upper face of the membrane, the images of the migrated cells to the lower surface were taken and cells were counted. Experiments were performed in triplicate.

### Xenograft studies

The use and handling of the animals conformed to the Turkish (26/4/2004-5199) and European (86/609/ECC) regulations, and were approved by the Institutional Ethics Committee. 4-6 week-old female SCID mice maintained under pathogen-free conditions were subcutaneously injected with 1.0x10^6^ cells / flank. While the right flanks of mice received the empty vector transfected or parental MDA-MB-231 cells as control, the left flanks of the animals were injected with SIK2 transfected cells (n=6 per group). 5 weeks post injections, tumors were excised from euthanized mice. The tumors were weighed and caliper measurements were used to estimate tumor volumes according to the formula: V = (L^*^W^2*^π)/6, where V: volume (mm^3^); L: largest diameter (mm); W: smallest diameter (mm).

### Bioinformatics analyses of target predictions, cell line and patient datasets

In ONCOMINE database screen, SIK2 transcript level and copy number were investigated in all fold changes and in all gene ranks within all measured genes, and p value was set as ^*^P<0.05. The data was obtained from 14 independent datasets that included different subtypes and grades of breast tumors with diverse clinical background.

Patient data were retrieved from the Molecular Taxonomy of Breast Cancer International Consortium (METABRIC) project with data deposited in EMBL European Genome–Phenome Archive (http://www.ebi.ac.uk/ega/) with an accession number EGAS00000000122) [37]; NCBI GEO databases GSE53752 [39], GSE21653 [42] and E-TABM-158 RNA sequencing dataset [40]. Gene names corresponding to probes which are used in the specific platforms are defined in each dataset. GSE53752 microarray data comprised of 51 triple-negative breast cancers, 25 normal breast tissues, and 106 luminal breast cancers. SIK2 expression level in TNBC (IDC) patients were compared with its expression in normal breast samples in the same dataset. In GSE21653 dataset consisting of basal and non-basal prognostic groups, patient survival was plotted based on their SIK2 gene expression profiles. In this dataset, the patients were separated as low against high SIK2 group, from 25th percentile. E-TABM-158 dataset includes tumor stage 2 (n=70) and 3-4 (n=19) breast tumors with normal tissue samples (n=26). KM plotter analysis was carried out for all, Luminal A, Luminal B and basal breast cancers regarding to mRNA level of SIK2 (kmplot.com/analysis/) [41]. For KM Plotter curves, ‘best cut-off’ option was selected for the separation of patients based on the level of SIK2 expression.

### Statistical analysis

Experimental groups were compared statistically using the Mann-Whitney test (one-tailed), one-way ANOVA or unpaired two-tailed student’s t-test. Significance of the differences in survival between two groups was calculated by Log-rank (Mantel-Cox) test. The results were presented as the means ± S.D. *P*<0.05 and *P*<0.001 were considered statistically significant.

### Ethics statement and consent to participate

The Ethics Committees of Bogazici University (Istanbul, Turkey) and the Florence Nightingale Hospital (Istanbul, Turkey) approved primary patient tissue study. The written informed consents were obtained from all participated patients. The use and handling of the animals conformed to the Turkish (26/4/2004-5199) and European (86/609/ECC) regulations, and were approved by the Bogazici University Institutional Ethics Committee.

## SUPPLEMENTARY MATERIALS FIGURES AND TABLES



## Abbreviations

ACTB: Beta-actin
Akt: Protein Kinase-B
CDH2: Cadherin-2
CIM: cell invasion/migration
CREB-1: CAMP responsive element binding protein 1
DFS: Disease-free survival
DMEM/F12: Dulbecco’s modified Eagle’s medium and Ham’s F12
DUSP4: Dual specificity protein phosphatase 4
EGF: Epidermal Growth Factor
EGFR: Epidermal Growth Factor Receptor
EMT: Epithelial-Mesenchymal Transition
ER/PR (+): Estrogen/Progesteron positive
ERK1/2: Extracellular signal-regulated protein kinases 1/2
FBS: Fetal Bovine Serum
FGF2: Fibroblast growth factor-2
Gab1: GRB2-associated-binding protein 1
HER2 (+): human epidermal growth factor receptor 2
HPRT: Hypoxanthine-guanine phosphoribosyltransferase
IDC: Invasive ductal carcinoma
IF: immunofluorescence
IHC: Immunohistochemistry
IR: Insulin receptor
IRS-1: Insulin receptor substrate-1
KRT-19: Keratin-19
MAPK: Mitogen-activated protein kinase
miRNA: MicroRNA
MMP-2: matrix metalloproteinase-2
MMP-9: matrix metalloproteinase-9
NEAA: Non-essential aminoacids
NF1: Neurofibromatosis type I
PBS: Phosphate-buffered saline
Pen/Str: Penicilin/Streptomycin
PFA: Paraformaldehyde
PI3K: phosphoinositide 3-kinase
PTEN: Phosphatase and tensin homolog
RFS: Relapse-free survival
RTCA: Real-Time Cell Analyzer
RTKs: Receptor tyrosine kinases
Sef: Similar expression to FGF genes
Ser/Thr: Serine/Threonine
SIK2: Salt-inducible Kinase2
SNAI2: Snail Family Transcriptional Repressor 2
Spry: Sprouty
TBS: Tris-buffered saline
TNBC: Triple-negative breast cancer
TSC1/2: Tuberous sclerosis 1/2
USP1: Ubiquitin carboxyl-terminal hydrolase 1
ZEB1: Zinc Finger E-Box Binding Homeobox 1
