# Supplementary material for: SIK2 attenuates proliferation and survival of breast cancer cells with simultaneous perturbation of MAPK and PI3K/Akt pathways
Source: Oncotarget. 2018 Apr 24;9(31):21876–92. doi: 10.18632/oncotarget.25082 (PMC5955149; doi:10.18632/oncotarget.25082)
Supplement: Supplementary file 1 [file oncotarget-09-21876-s001.pdf]

SIK2 attenuates proliferation and survival of breast cancer cells with simultaneous perturbation of MAPK and PI3K/Akt pathways

SUPPLEMENTARY MATERIALS

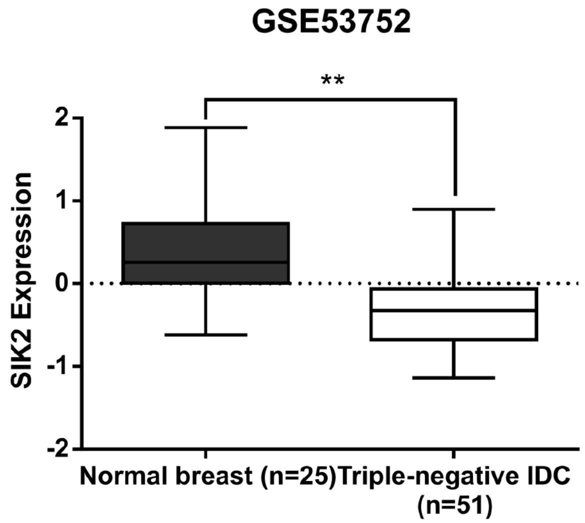

Supplementary Figure 1: SIK2 Expression in Triple Negative IDC patients. In GSE53752 dataset which has the expression data from triple-negative IDC patients and normal breast tissues revelaed a lower SIK2 expression in triple-negative IDCs (n=51) as compared to normal breast tissue (n=25). Statistical significance was assesed by unpaired, two-tailed student t-test. (**, *P*<0.01).

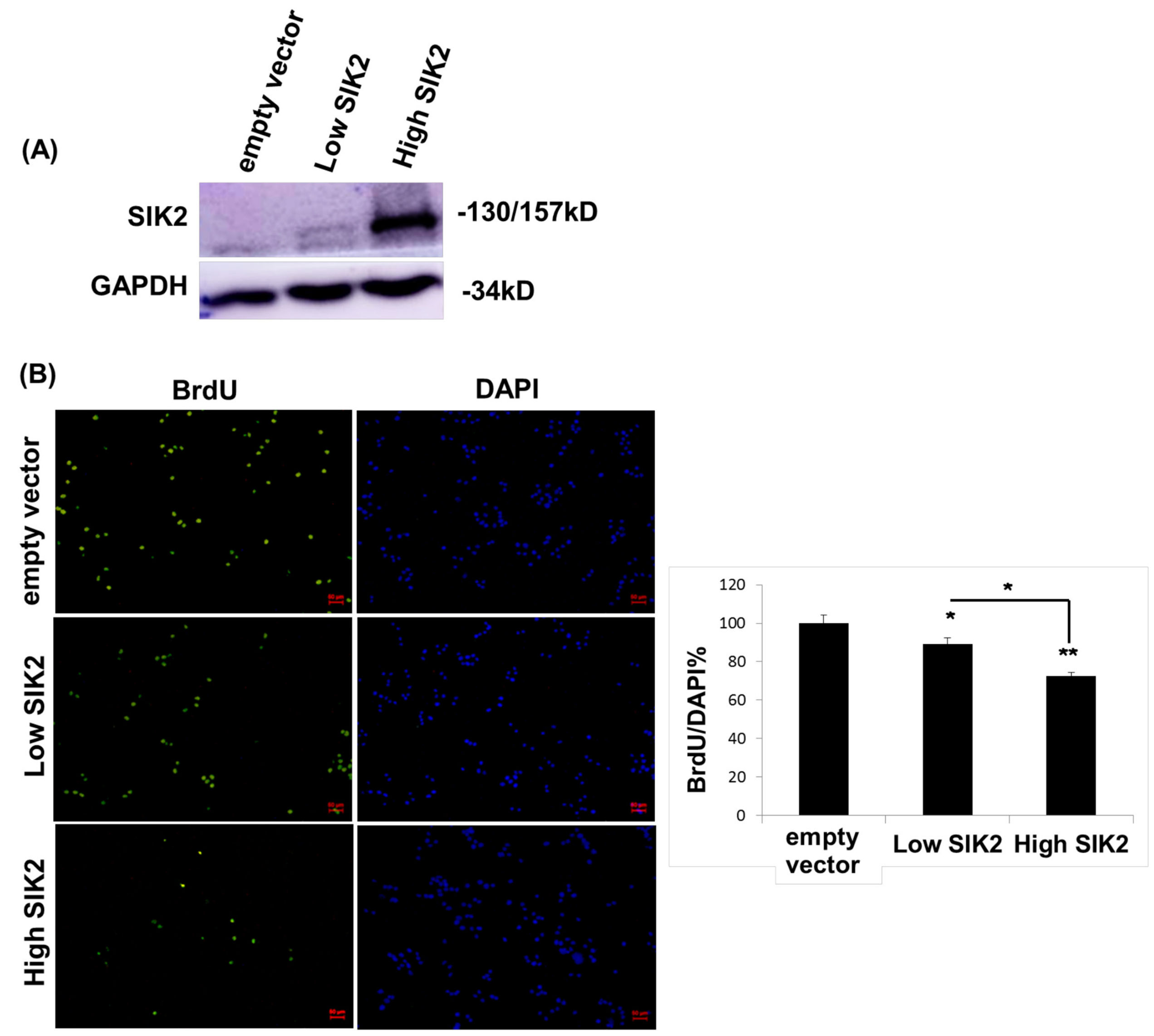

Supplementary Figure 2: Dose-dependent SIK2 overexpression leads to dose-dependent growth inhibition of MDA-MB-231 cells. (A) SIK2 expression was modulated in MDA-MB-231 cells by transfection with full-length SIK2. Control cells were transfected with empty vector. Changes in SIK2 levels in two different clones of SIK2 OE cells (Low SIK2, c1 and high SIK2, c2), compared to empty vector-transfected cells were evaluated by Western blotting. SIK2 band intensities were normalized to that of GAPDH in the same samples. **(B)** Proliferation was assessed by BrdU incorporation assay. Bar graphs represent the mean values of at least 3 independent biological samples (± SD). In BrdU assay, cell nuclei were visualized by DAPI staining and in each sample at least 150-200 cells were counted, the scale bar=50um. $^{**}P< 0.001$, and $^{*}P\leq 0.05$.

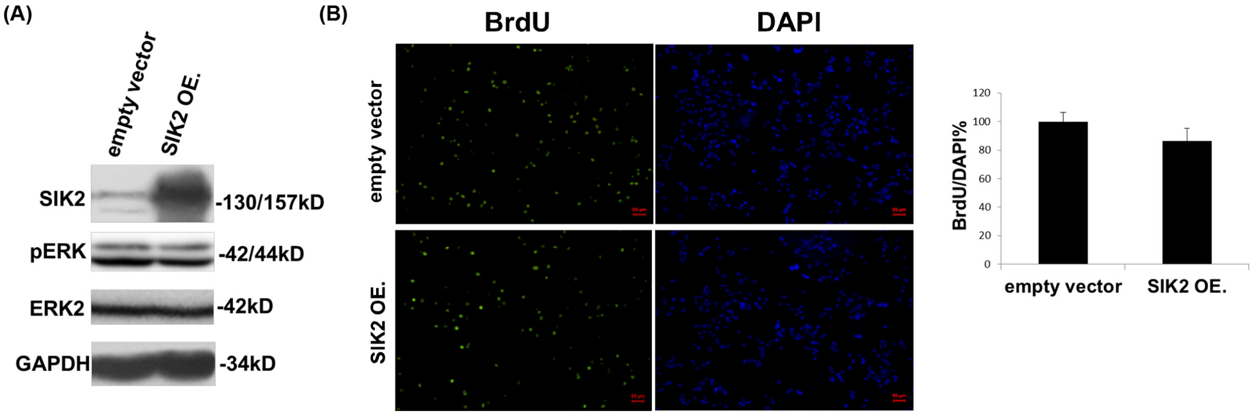

Supplementary Figure 3: SIK2 upregulation does not reduce the cell growth and Erk phosphorylation in SK-BR-3 cells. (A) SIK2 expression was modulated in SKBR3 cells by transfection with full-length SIK2. Control cells were transfected with empty vector. Changes in SIK2 levels compared to empty vector transfected cells were evaluated by Western blotting. SIK2 band intensities were normalized to that of GAPDH in the same samples. **(B)** Proliferation was assessed by BrdU incorporation assay. Bar graphs represent the mean values of at least 3 independent biological samples (± SD). In BrdU assay, cell nuclei were visualized by DAPI staining and in each sample at least 150-200 cells were counted, the scale bar=50um.

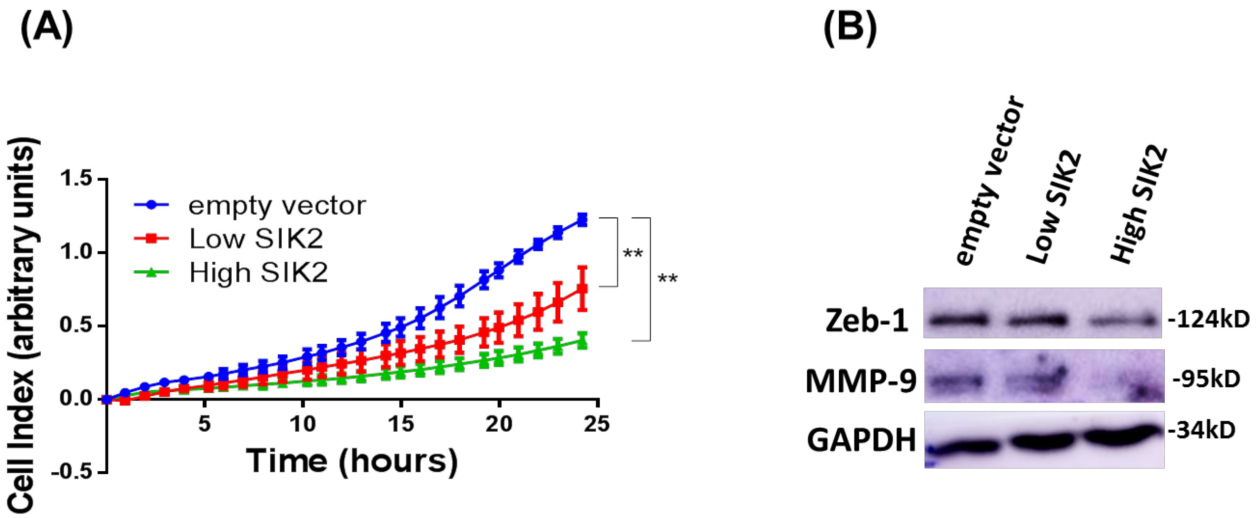

Supplementary Figure 4: SIK2 overexpression blocks the migration of MDAMB231 cells in a dose-dependent manner. **(A)** Effect of dose-dependent upregulation of SIK2 on the migration capacity of MDA-MB-231 cells was analyzed by RTCA experiment in a 24 hour time frame. **(B)** The effect of dose-dependent increase in SIK2 levels on ZEB1 and MMP-9 expression profiles was studied by Western blot analysis and anti-GAPDH antibody was used as a loading control. All experiments were done in triplicates. $^{**}P< 0.001$.

Supplementary Table 1: Oncomine database screening

(A) Oncomine database screen for sik2 expression. Oncomine database was screened for SIK2 transcript level and 13 independent breast cancer datasets were retrieved.

Data sample/ publication/ total genes measured	Total samples normal/ tumors verified	Transcript level	Fold change	Significance (P-Value < 0.05)
Curtis Breast/Nature/ 2012/04/08 19.273 genes	144/1992	Decrease	-1.616	3.51E-21
Sorlie Breast/ Proc Natl Acad Sci U S A 2001/09/11/ 6304 genes	4/79	Decrease	-3.13	5.67E-08
TCGA Breast Nature/2012/ 20423 genes	61/532	Decrease	-3.024	1.06E-17
Perou Breast/Nature 2000/08/17/6625 genes	4/59	Decrease	-2.528	1.38E-05
Sorlie Breast/Proc Natl Acad Sci U S A 2003/07/08/6,197 genes	3/138	Decrease	-3.141	1.90E-04
Finak Breast/Nat Med 2008/05/01/19,189 genes	6/53	Decrease	-8.032	3.44E-08
Richardson Breast/Cancer Cell 2006/02/01/19,574 genes	7/40	Decrease	-2.281	1.58E-08
Gluck Breast/Breast Cancer Res Treat 2011/03/04/17.862 genes	4/154	Decrease	-2.782	0.032
Ma Breast 4/Breast Cancer Res 2009/02/02/19,139 genes	28/38	Decrease	-2.864	5.28E-4
Karnoub Breast/Nature 2007/10/04/19.574 genes	15/7	n.s. change	-1.561	0.079
Zhao Breast/Mol Biol Cell 2004/06/01/12,482 genes	3/58	n.s. change	-2.061	0.070
Radvanyi Breast/Proc Natl Acad Sci U S A 2005/08/02/16,775 genes	5/21	n.s. change	-1.482	0.303
Turashvili Breast/BMC Cancer/200719.574 genes	20/30	n.s. change	-1.224	0.209

(B) Oncomine database screen for DNA copy number. TCGA Breast (2) dataset (Nature, 2012) was screened for SIK2 gene copy number. All screenings were done using filtering criteria for all fold changes and in all gene ranks within all measured genes, and p-values were set to ≤0.05.

Data sample/ publication/total genes measured	Total samples normal/tumor verified	DNA copy number	Fold change	Significance (P-Value < 0.05)
TCGA Breast 2/No Associated Paper 2012/02/29/18,823 genes	813/759	Decrease	-1.199	5.53E-05

Supplementary Table 2: Histopathological features of breast cancer patients

Patient No	Diagnosis*	ER/PR status	ErbB2 status	Histological grade**	Proliferation index (%)	Prevalence of SIK2 staining	
						Tumor	Normal
P1	IDC	ER/PR(+)	+	1	4	4	4
P2	IDC	ER/PR(+)	+	3	30	1	4
P3	IDC	ER/PR(+)	+	2	15	1	4
P4	IDC	ER/PR(+)	+	2	15	1	4
P5	IDC	ER/PR(+)	+	2	5	4	4
P6	IDC	ER/PR(+)	+	2	30	1	4
P7	IDC	ER/PR(+)	+	2	12	4	4
P8	IDC	ER/PR(+)	+	3	25	1	4
P9	IDC	ER/PR(+)	-	2	25	1	4
P10	IDC	ER/PR(+)	-	2	20	1	4
P11	IDC	ER/PR(+)	-	3	30	2	4
P12	IDC	ER/PR(+)	-	2	5	4	4
P13	IDC	ER/PR(+)	-	1	5	4	4
P14	IDC	ER/PR(+)	-	2	10	4	4
P15	IDC	ER/PR(+)	-	2	15	1	4
P16	IDC	ER/PR(+)	-	2	10	4	4
P17	IDC	ER/PR(+)	-	2	10	4	4
P18	IDC	ER/PR(+)	-	1	5	0	4
P19	IDC	TNBC	TNBC	3	80	2	4
P20	IDC	TNBC	TNBC	3	70	0	4
P21	IDC	TNBC	TNBC	3	70	0	4
P22	IDC	TNBC	TNBC	3	35	0	4
P23	IDC	TNBC	TNBC	3	60	0	4
P24	IDC	TNBC	TNBC	2	50	0	4
P25	IDC	TNBC	TNBC	3	40	1	4
P26	IDC	TNBC	TNBC	3	45	1	4
P27	IDC	TNBC	TNBC	3	60	0	4
P28	IDC	TNBC	TNBC	2	50	0	4
P29	IDC	TNBC	TNBC	2	15	1	4
P30	IDC	TNBC	TNBC	2	30	0	4

The table summarizes the histopathological features of tumor and normal samples from primary breast cancer patients (n=30). ER/PR/Her-2 status, Ki67 level and grades of the tumors were evaluated by pathological analysis. In the same samples, extent of SIK2 expression was also studied by immunofluorescent staining.

*: Invasive Ductal Carcinoma.

**: Nottingham Grade Histologic Combined System.

Supplementary Table 3: List of primers used in RT-PCR

Gene ID	Forward primer	Reverse primer
SIK2	5'-CTGGACATCTGGAGTATGGG-3'	5'-GAATCTTCCTTCCAGAACCCT-3'
EPCAM	5'-CGCAGCTCAGGAAGAATGTG-3'	5'-TGAAGTACACTGGCATTGACG-3
CLAUDIN-7	5'-CCACTCGAGCCCTAATGGTG -3'	5'-GGTACCCAGCCTTGCTCTCA-3'
KRT-19	5'-CTTCCGAACCAAGTTTGAGAC-3'	5'-GAATCCACCTCCACACTGAC-3'
CDH2	5'-ACAGTGGCCACCTACAAAGG-3'	5'-CCGAGATGGGGTTGATAATG-3'
FN	5'-CTGGCCGAAAATACATTGTAAA-3'	5'-CCACAGTCGGGTCAGGAG-3'
SNAI2	5'-TGGTTGCTTCAAGGACACAT-3'	5'-GTTGCAGTGAGGGCAAGAA-3'
ZEB1	5'-GGGAGGAGCAGTGAAAGAGA-3'	5'-TTTCTTGCCCTTCCTTTCTG-3'
ZEB2	5'-AAGCCAGGGACAGATCAGC-3'	5'-CCACACTCTGTGCATTTGAACT-3'
ACTB	5'-CCAACCGCGAGAAGATGA-3'	5'-CCAGAGGCGTACAGGGATAG-3'
HPRT	5'-TGACCTTGATTTATTTTGCATACC-3'	5'-CGAGCAAGACGTTCAGTCCT-3'
GAPDH	5'-GCCCAATACGACCAAATCC-3'	5'-AGCCACATCGCTCAGACAC-3'

Supplementary Table 4: Antibodies used in Western blotting and IF/IHC staining

Protein ID	Firm	Catalog number	Dilution
phospho-AKT (Ser473)	Cell Signaling Technology	CST4060S	1/1000
Total-AKT	Cell Signaling Technology	CST9272	1/1000
GAPDH	Cell Signaling Technology	CST5174	1/1000
Vimentin	Cell Signaling Technology	CST5741	1/1000
Beta-actin	MP Biomedicals	69100	1/10000
E-Cadherin	Abcam	32A8	1/1000
SIK2	Novus Biologicals	NBP42690	1/1000
SIK2	Cell Signaling Technology	CST6919	1/1000
phospho ERK	Cell Signaling Technology	CST4370	1/1000
Total ERK	Cell Signaling Technology	CST9102	1/1000
MMP-2	Abcam	D8N9Y	1/1000
MMP-9	Abcam	ab58803	1/1000
Cleaved caspase-3	Cell Signaling Technology	CST9661	1/1000
Ki67	BD Biosciences	BD550609	1/250
beta actin HRP	Santa Cruz	sc-47778	1/10000
mouse HRP	Santa Cruz	sc-2005	1/10000
rabbit HRP	Santa Cruz	sc-2004	1/10000
Slug	Abcam	ab27568	1/1000
ZEB-1	Santa Cruz	sc-25388	1/1000
Alexa-Fluor-488	Invitrogen	A-31572	1/250
Alexa-Fluor-555	Invitrogen	A-21202	1/250
DAPI	Sigma Aldrich	10236276001	1/10000
